# Biomechanical signaling within the developing zebrafish heart attunes endocardial growth to myocardial chamber dimensions

**DOI:** 10.1038/s41467-019-12068-x

**Published:** 2019-09-11

**Authors:** Dorothee Bornhorst, Peng Xia, Hiroyuki Nakajima, Chaitanya Dingare, Wiebke Herzog, Virginie Lecaudey, Naoki Mochizuki, Carl-Philipp Heisenberg, Deborah Yelon, Salim Abdelilah-Seyfried

**Affiliations:** 1 Institute of Biochemistry and Biology, Potsdam University, D-14476 Potsdam, Germany; 2 Institute of Molecular Biology, Hannover Medical School, D-30625 Hannover, Germany; 3 Institute of Science and Technology Austria, 3400 Klosterneuburg, Austria; 4 Department of Cell Biology, National Cerebral and Cardiovascular Center Research Institute, Osaka, 565-8565 Japan; 5 Institute of Cell Biology and Neuroscience, Department of Developmental Biology of Vertebrates, Goethe Universität Frankfurt am Main, 60438 Frankfurt am Main, Germany; 6 Max Planck Institute for Molecular Biomedicine, 48149 Münster, Germany; 7 Cells-in-Motion Cluster of Excellence (EXC 1003-CiM), University of Münster, 48149 Münster, Germany; 8 AMED-CREST. National Cerebral and Cardiovascular Center, Osaka, 565-8565 Japan; 9 Division of Biological Sciences, University of California, San Diego, La Jolla, CA 92093 USA

**Keywords:** Cell biology, Morphogenesis

## Abstract

Intra-organ communication guides morphogenetic processes that are essential for an organ to carry out complex physiological functions. In the heart, the growth of the myocardium is tightly coupled to that of the endocardium, a specialized endothelial tissue that lines its interior. Several molecular pathways have been implicated in the communication between these tissues including secreted factors, components of the extracellular matrix, or proteins involved in cell-cell communication. Yet, it is unknown how the growth of the endocardium is coordinated with that of the myocardium. Here, we show that an increased expansion of the myocardial atrial chamber volume generates higher junctional forces within endocardial cells. This leads to biomechanical signaling involving VE-cadherin, triggering nuclear localization of the Hippo pathway transcriptional regulator Yap1 and endocardial proliferation. Our work suggests that the growth of the endocardium results from myocardial chamber volume expansion and ends when the tension on the tissue is relaxed.

## Abstract (web summary)

It is unknown how endocardium growth is coordinated with that of the myocardium in the zebrafish. Here, the authors show that myocardial chamber volume expansion causes increased endocardial tissue tension, which in turn triggers Hippo signaling-mediated proliferation within the endocardium.

## Introduction

The growth and morphogenesis of organs requires some means of intra-organ communication between its different tissues. In the heart, biochemical signaling of secreted factors such as Wnts, BMPs, FGFs, or components of the extracellular matrix and cell–cell communication via the Notch, Neuregulin/ErbB2, or Ephrin signaling pathways^1–3^ are involved. Yet, it is unknown which modes of communication coordinate the growth of the endocardium with that of the myocardium. In zebrafish, during the process of cardiac ballooning at 30–54 hours post fertilization (hpf), endocardial chambers grow by proliferation with neither accretion of cells from external sources, nor from cellular intermingling over the chamber boundary at the atrioventricular canal^4^. In striking contrast, the myocardium grows mostly through an accretion of cells to the chamber poles^5–8^ and due to cell size increases^9,10^. Here, we use two different genetic conditions (loss of Nkx2.5 or increase in Wnt8a expression) that cause an exaggerated expansion of myocardial atrial chamber dimensions. Comparing these two extreme conditions to WT reveals that an increased expansion of myocardial atrial chamber dimensions is compensated by increased endocardial proliferation and cell numbers. We find that an increased expansion of the atrial chamber volume generates higher junctional forces within endocardial cell junctions which are transduced by the endothelial-specific adherens junction protein Cadherin-5 (VE-cadherin) to activate Hippo pathway transcriptional regulator Yap1-dependent endocardial proliferation.

## Results

### Endocardial cell proliferation increases in larger atrial chambers

To examine whether changes in the dimensions of the myocardial chambers affect endocardial cell numbers, we analyzed two well-established genetic conditions that cause changes in their relative dimensions. Upon heat shock at 24 hpf, the atrial myocardium of the zebrafish transgenic line *Tg(hsp70l:wnt8a-GFP)*^*w34*^ (referred to as *hs:wnt8a*) undergoes a massive increase in volume and cell numbers as determined at 52 hpf, whereas the ventricular chamber decreases in size^11^. We also employed the *nkx2*.*5*^*vu179*^ mutant, which causes a comparable myocardial phenotype^12^. In both genetic conditions, atrial chamber growth involves increases in myocardial cell numbers while numbers of ventricular cardiomyocytes are reduced (Supplementary Fig. 1)^11,13,14^. We reasoned that these two different genetic conditions would be good tools to study the response of the endocardium to this mode of myocardial atrial chamber expansion.

**Fig. 1:**
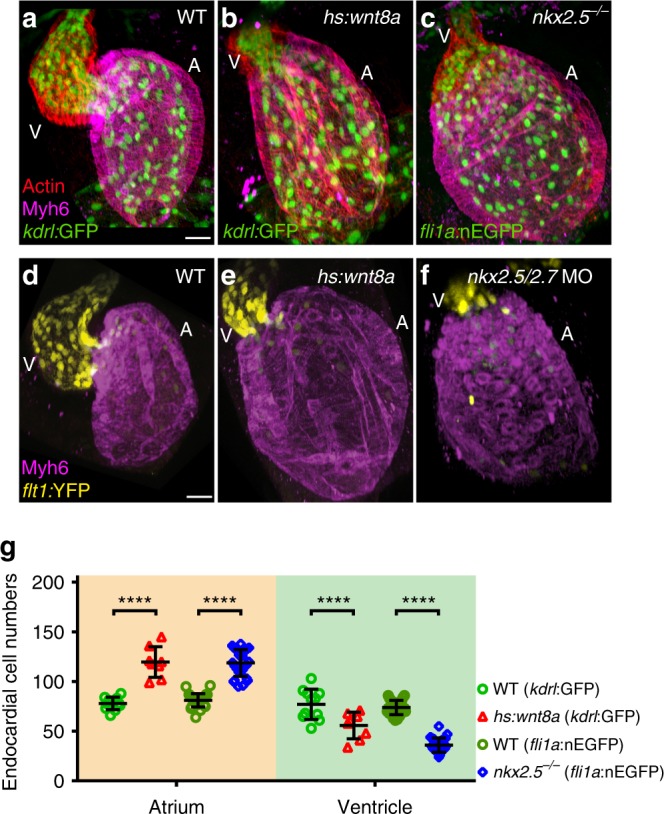
Overexpression of Wnt8a or loss of Nkx2.5 causes a shift in endocardial chamber proportions. **a**–**c** Reconstruction of confocal z-stacks show the endothelial-specific transgenic reporter lines *Tg(kdrl:EGFP)*^s843^ or *Tg(fli1a:nEGFP)*^*y7*^ (green), Phalloidin 568-stained Actin (red) and anti-Myh6 labeling of the myocardial atrial chamber (magenta). Compared to (**a**) WT, (**b**) overexpression of Wnt8a, or (**c**) the *nkx2*.*5*^*vu179*^ mutation, causes a relative shift of endocardial chamber dimensions. **d**–**f** Reconstructions of confocal z-stacks shows that the arterial endothelial transgene *Tg(flt1:YFP)*^*hu4624*^ marks the ventricular chamber whereas the myocardial atrial marker anti-Myh6 labels the atrial chamber. Compared to (**d**) WT, (**e**) overexpression of Wnt8a, or (**f**) loss of Nkx2.5/Nkx2.7 results in relative shifts of chamber dimensions as indicated by the ventricular-specific expression of *Tg(flt1:YFP)*^*hu4624*^ within the endocardium. A, atrium; V, ventricle. Scale bars, 30 μm. **g** Quantifications of endocardial cell numbers in WT (*kdrl*:GFP *n* = 11 hearts, *fli1a*:nEGFP *n* = 27 hearts), *Tg(hsp70l:wnt8a-GFP)*^*w34*^ (*n* = 8 hearts), or *nkx2*.*5*^*vu179*^ mutants (*n* = 27 hearts) reveal that atrial endocardial cell numbers are significantly increased and ventricular endocardial cell numbers are significantly reduced compared to WT. Mean values ± SD are shown. Two-way ANOVA was used to compare each condition with its WT control in each individual chamber (****p < 0.0001)

We found that under these two genetic conditions, the dimensions of the endocardial chambers underwent comparable changes at 52 hpf, which is the stage when  relative chamber dimensions are established. Compared with WT, the overexpression of Wnt8a resulted in a relative shift of endocardial chamber dimensions with an enlarged atrium containing higher endocardial cell numbers and a smaller ventricle (Fig. 1a, b, g). To clearly identify chamber identities, the transgenic reporter line *Tg(flt1:YFP)*^*hu4624*^ which mainly marks ventricular endocardial cells^15^ was counter-stained with the myocardial atrial marker anti-Myosin heavy chain 6 (Myh6) which includes cells at the atrioventricular canal where Myh6-positive cardiomyocytes form a sharp boundary (Fig. 1d, e). Similarly, relative shifts in chamber dimensions with an increase of atrial endocardial cell numbers occurred in *nkx2*.*5*^*vu179*^ mutants (Fig. 1c, g)^13,14^ which are phenotypically similar to the antisense oligonucleotide morpholino (MO)-mediated knock down of Nkx2.5/Nkx2.7^13^. The latter condition resulted in a smaller endocardial ventricular chamber, as indicated by the expression of *Tg(flt1:YFP)*^*hu4624*^ (Fig. 1f). These endocardial-specific changes mirrored those that occur within the myocardium under these conditions (Supplementary Fig. 1)^11–13^.

**Fig. 2:**
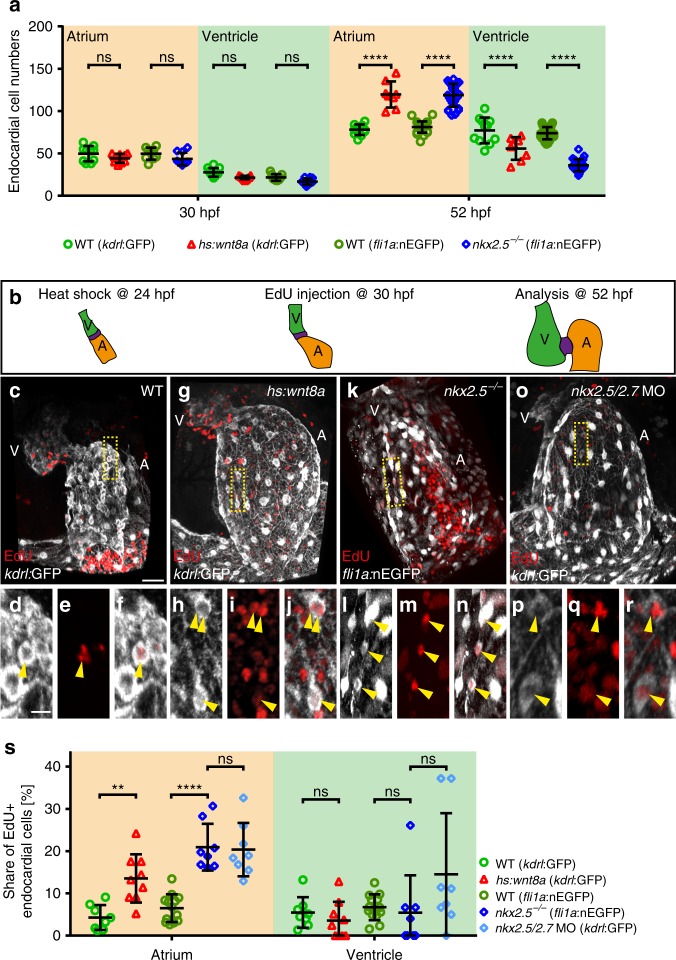
Atrial endocardial cell proliferation is increased upon Wnt8a overexpression or in *nkx2*.*5*^*vu179*^ mutants. **a** Quantifications of endocardial cell numbers at 30 hpf reveals that there is no increase of atrial endocardial cells in *Tg(hsp70l:wnt8a-GFP)*^*w34*^ (*n* = 9 hearts), or *nkx2*.*5*^*vu179*^ mutant embryos (*n* = 10 hearts) compared with WT (*kdrl*:GFP *n* = 10 hearts, *fli1a*:nEGFP *n* = 9 hearts) at this stage. At the later heart ballooning stage (52 hpf), a significant increase of endocardial atrial cell numbers has occurred upon Wnt8a overexpression [*Tg(hsp70l:wnt8a-GFP)*^*w34*^ (*n* = 8 hearts)] or in *nkx2*.*5*^*vu179*^ mutants (*n* = 27 hearts) compared to WT (*kdrl*:GFP *n* = 11 hearts, *fli1a*:nEGFP *n* = 27 hearts). Mean values ± SD are shown. Two-way ANOVA was used to compare each condition with its WT control in each individual chamber (ns: not significant,****p < 0.0001). **b** Schematic model of the EdU assay used. WT and *Tg(hsp70l:wnt8a-GFP)*^*w34*^ transgenic embryos were heat shocked at 24 hpf. Embryos of all genotypes were injected with EdU into the circulatory system at 30 hpf and analyzed at 52 hpf. **c**, **g**, **k**, **o** Reconstructions of confocal z-stacks showing representative hearts at 52 hpf of **(c**) WT, (**g**) upon Wnt8a overexpression, (**k**) in *nkx2*.*5*^*vu179*^ mutants, or (**o**) in *nkx2*.*5*/*nkx2*.*7* double morphants. Endocardial tissue is marked by *Tg(kdrl:EGFP)*^*s843*^ or *Tg(fli1a:nEGFP)*^*y7*^ reporters (white) and proliferative cells are marked by EdU incorporation (red). A, atrium; V, ventricle. Scale bars, 30 μm. **d**–**f**, **h**–**j**, **l**–**n**, **p**–**r** Shown are magnifications of insets (yellow boxes) with *kdrl*:GFP or *fli1a*:nEGFP/EdU double positive cells (yellow arrowhead). Single channel with *kdrl*:GFP or *fli1a*:nEGFP (**d**, **h**, **l**, **p**), EdU incorporation (**e**, **i**, **m**, **q**) and the merge of both channels (**f**, **j**, **n**, **r**). Scale bars, 10 μm. **s** Quantifications of the share of EdU + atrial or ventricular endocardial cells relative to the total number of endocardial cells within the respective cardiac chamber in WT (*kdrl*:GFP *n* = 8 hearts, *fli1a*:nEGFP *n* = 13 hearts), upon Wnt8a overexpression (*n* = 9 hearts), *nkx2*.*5*^*vu179*^ mutants (*n* = 8 hearts), or *nkx2*.*5*/*nkx2*.*7* double morphants (*n* = 8 hearts). Upon Wnt8a overexpression or loss of Nkx2.5 or Nkx2.5/Nkx2.7, proliferation significantly increases in the developing atrial endocardium. Mean values ± SD are shown. Two-way ANOVA was used to compare each condition with its WT control in each individual chamber (ns: not significant; ***p < 0.001, ****p < 0.0001)

One explanation for the increased number of endocardial cells might be their proliferation. We found that the numbers of atrial and ventricular endocardial cells were identical between all genetic conditions analyzed at 30 hpf (Fig. 2a). This indicates that the sizes of endocardial progenitor cell pools were not altered. Hence the respective proliferation rates were examined by injections of EdU into the circulatory system at 30 hpf and analyzed its incorporation within endocardial cells at 52 hpf (Fig. 2b). As a result, the rate of endocardial cell proliferation increased significantly within the atrium but not the ventricle compared to WT (Fig. 2c–f, s) upon Wnt8a overexpression (Fig. 2g–j, s), in *nkx2*.*5*^*vu179*^ mutants (Fig. 2k–n, s), or upon knock down of Nkx2.5/Nkx2.7 (Fig. 2o–r, s). In contrast, no increase in cell proliferation was observed upon Wnt8a overexpression within the myocardium despite strong EdU-labeling within some extra-cardiac tissues (Supplementary Fig. 2). This is similar to what has been reported for *nkx2*.*5*^*vu179*^ mutants or *nkx2*.*5*/*nkx2*.*7* double morphants^12,13^. Hence, the increases in atrial endocardial cell numbers correspond with more proliferation.

### Endocardial cell sizes are unaltered in enlarged atrial chambers

**Fig. 3:**
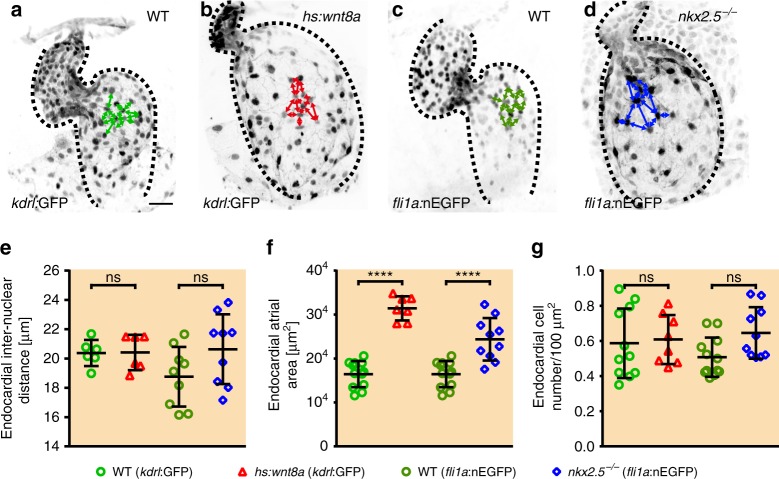
Endocardial cell sizes are not altered upon atrial chamber expansion. **a**–**d** Reconstructions of confocal z-stacks of representative hearts at 52 hpf of (**a**) WT, (**b**) upon Wnt8a overexpression, (**c**) WT, or (**d**) in *nkx2*.*5*^*vu179*^ mutants. Endocardial GFP expression of *Tg(kdrl:EGFP)*^*s843*^ or *Tg(fli1a:nEGFP)*^*y7*^ is inverted in black/white. Endocardial inter-nuclear distances are indicated by arrows and a dotted line indicates the outline of the endocardium. Scale bars, 30 µm. **e** Quantifications of endocardial inter-nuclear distances reveal no significant differences between WT (*kdrl*:GFP *n* = 6 hearts, *fli1a*:nEGFP *n* = 9 hearts), upon Wnt8a overexpression (*n* = 6 hearts), or in *nkx2*.*5*^*vu179*^ mutants (*n* = 9 hearts). Each dot represents one heart with an average of at least 20 length measurements within the endocardial atrium. **f** Quantifications of the atrial endocardial surface area reveals a significant increase upon Wnt8a overexpression (*n* = 7 hearts) or loss of Nkx2.5 (*n* = 10 hearts) compared to WT hearts (*kdrl*:GFP *n* = 12 hearts, *fli1a*:nEGFP *n* = 12 hearts). **g** Quantifications of the ratio of endocardial cell numbers relative to total atrial chamber area indicates no significant differences between WT (*kdrl*:GFP *n* = 11 hearts, *fli1a*:nEGFP *n* = 12 hearts), upon Wnt8a overexpression (*n* = 8 hearts), or in *nkx2*.*5*^*vu179*^ mutants (*n* = 10 hearts). **e**–**g** Mean values ± SD are shown. One-way ANOVA was used to compare each condition with its WT control (*ns*: not significant, ****p < 0.0001)

Using an in vivo multi-color mosaic labeling technique^16^ and morphometric measurements of immunolabeled hearts, we discovered that increased Wnt8a expression or loss of Nkx2.5 caused striking increases in the size of atrial cells within the myocardium (Supplementary Fig. 3 a–f; Supplementary Movies 1,2). We also used an alternative measurement and determined inter-nuclear distances in WT and upon Wnt8a overexpression, which provided the same result (Supplementary Fig. 3g). We wondered whether endocardial cell sizes were equally affected by the strong expansion of cardiac chamber dimensions. To quantify endocardial cell sizes, we measured at least 20 endocardial inter-nuclear distances per heart using the endocardial marker lines *Tg(kdrl:EGFP)*^*s843*^ or *Tg(fli1a:nEGFP)*^*y7*^ at 52 hpf. Although atrial endocardial chambers were expanded under these conditions, Wnt8a overexpression (Fig. 3b, e–g) or loss of Nkx2.5 (Fig. 3d–g) did not result in significant changes in inter-nuclear distances or endocardial cell densities within the atrial endocardium compared to WT (Fig. 3a, c, e–g). Thus cell size increases are not responsible for the expansion of the atrial endocardial chamber in these two conditions.

### Endocardial cell junctional tension increases within a larger atrium

Our observations suggested that myocardial chamber dimensions influence the proliferation of endocardial cells. This hinted at some form of intra-organ communication between the myocardium and endocardium. Cells within the endocardium and myocardium are separated by a layer of extracellular matrix (cardiac jelly)^2^ which can propagate mechanical tension^17,18^. We hypothesized that when the size of endocardial cells remains constant, tensile forces on their cell junctions rise through the expansion of atrial chambers that occurs upon Wnt8a overexpression or loss of Nkx2.5/Nkx2.7.

**Fig. 4:**
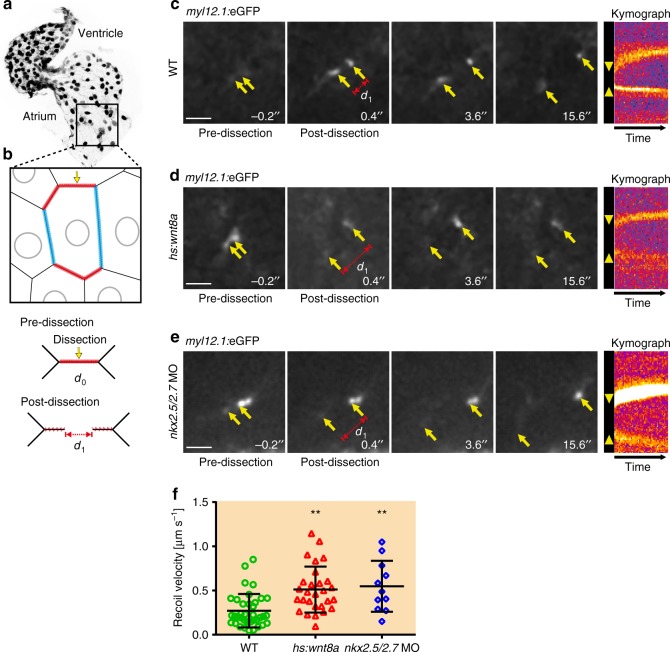
Endocardial tissue tension increases upon Wnt8a overexpression or loss of Nkx2.5/Nkx2.7. **a**, **b** Schematic model illustrating the endocardial region in which the laser cuts were performed. Local laser cut (yellow arrow) of the subcortical actomyosin network within the shorter membrane compartment (red) which is oriented perpendicular to the direction of intra-cardiac blood flow at time point d_0_ causes an actomyosin recoil (d_1_). **c**–**e** Time lapse sequences following a laser cut within the endocardium of 40 hpf embryos with the *Tg(act2:myl12*.*1-EGFP)*^*e2212*^ transgenic reporter that marks the actomyosin network. Yellow arrows show the actomyosin recoil distance within 0.4 ms upon laser dissection. Kymographs indicate the temporal recoil of the actomyosin network along the membrane compartment where the laser cut was performed. The recoil of the open junction ends is visualized with the fluorescence intensity and the initial opening of the junction is marked by yellow arrowheads. **d**, **e** Time lapse analyses demonstrate a larger opening of the junction (d_1_) and a faster recoil of the actomyosin network (yellow arrowheads in the kymograph) upon Wnt8a overexpression or loss of Nkx2.5/Nkx2.7 following laser cuts. Scale bars, 20 μm. **f** Comparison of initial recoil velocities (μm per sec) which are lower in WT (*n* = 37 hearts) compared with Wnt8a overexpressing embryos (*n* = 28 hearts) or upon loss of Nkx2.5/Nkx2.7 (*n* = 11 hearts). Mean values ± SD are shown. One-way ANOVA was used to compare each condition with WT (**p < 0.001)

To address this issue, we adapted an ultraviolet (UV)-laser dissection set-up^19^ to measure cell junctional forces within the zebrafish endocardium. The transgenic reporter line *Tg(act2:myl12*.*1-EGFP)*^*e2212*^, which labels the non-muscle myosin II cytoskeleton, distinctly marks the sub-membranous compartment of endocardial cells^20^ which is distinct from the sarcomeric myocardium (Supplementary Movie 3). This permitted us to direct the UV-laser at cell junctions to measure the recoil of endocardial cell junctions upon dissection. To assess whether cell junction tensile forces differed between blood flow-parallel and cell junctions with an orientation perpendicular to the direction of blood flow, we performed measurements of recoil velocities in WT. This revealed no significant differences in initial recoil velocities between these different membrane compartments (Supplementary Fig. 4). To compare cell junctional forces, we targeted only the shorter perpendicular cell junction compartments of endocardial cells (red membrane compartments in Fig. 4a, b) and performed only one dissection per endocardium within a cell positioned near the inflow tract region to avoid tissue-relaxation upon neighboring cells (Fig. 4a, b).

To assess junctional tensile forces while the chamber expansion process was still ongoing at 40 hpf, we anaesthetized embryos and paused their heart beat with tricaine, which does not affect non-muscle myosin II. Time lapse movies of *Tg(act2:myl12*.*1-EGFP)*^*e2212*^ localization after the cell junction cuts were recorded for a period of 15.6 s at 0.2 second intervals (Fig. 4c–e; Supplementary Movies 4-6). Upon Wnt8a overexpression, tensile forces in endocardial cell junctions are higher compared with WT (Fig. 4c), as indicated by a greater width of myosin recoil at 0.4 s after laser dissection (Fig. 4d). Based on slope measurements of the kymographs (see Materials and Methods), we determined the initial recoil velocities (μm per sec) in WT (Fig. 4c), upon Wnt8a overexpression (Fig. 4d), or upon loss of Nkx2.5/Nkx2.7 (Fig. 4e). These analyses revealed that endocardial cell junctions are under higher tensile forces while chamber dimensions are expanding (Fig. 4f). Our findings imply that the expanding dimensions of atrial myocardial chambers cause stronger tensile forces upon cell junctions within the endocardium. This finding suggested that in response, endocardial cells may activate biomechanical signaling pathways to regulate proliferation, which subsequently releases tension within this tissue.

### Cadherin-5 mediates endocardial proliferation during chamber expansion

**Fig. 5:**
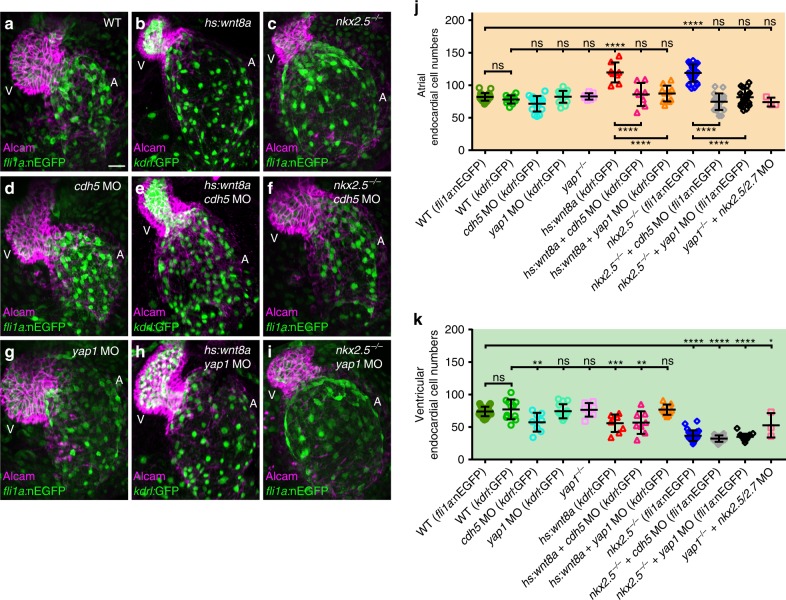
Loss of Cadherin-5 or Yap1 prevents endocardial cell number increases during atrial chamber expansion. **a–i** Reconstructions of confocal z-stacks of zebrafish hearts at 52 hpf expressing the endocardial reporter *Tg(kdrl:EGFP)*^*s843*^ or *Tg(fli1a:nEGFP)*^*y7*^ (green) and immunolabeling against the myocardial marker Alcam (magenta). **j** Quantifications of endocardial cell numbers at 52 hpf. **b**, **j** Upon Wnt8a overexpression (*n* = 7 hearts), atrial endocardial cell numbers increase. **c**, **j** Similarly, in *nkx2*.*5*^*vu179*^ mutants (*n* = 27 hearts), endocardial cell numbers increase within the atrium. **d**, **j** MO-mediated knock down of Cadherin-5 (Cdh5) (*n* = 7 hearts) does not cause a reduction of endocardial cell numbers within the atrium. **e**, **j** Loss of Cdh5 prevents increased atrial endocardial cell numbers upon overexpression of Wnt8a (*n* = 8 hearts). **f**, **j** Loss of Cdh5 suppresses increased atrial endocardial cell numbers in *nkx2*.*5*^*vu179*^ mutants (*n* = 19 hearts). **g**, **j** Loss of Yap1 via MO-mediated knock down (*n* = 14 hearts) or in *yap1*^*fu48*^ mutants (**j**, **k**) does not affect ventricular or atrial endocardial cell numbers. **h**, **j** Knock down of Yap1 in Wnt8a overexpressing embryos normalizes ventricular and atrial endocardial cell numbers (*n* = 8 hearts). **i**, **j** Loss of Yap1 rescues atrial endocardial cell numbers in *nkx2*.*5*^*vu179*^ mutants (*n* = 17 hearts). **j** A loss of Nkx2.5/Nkx2.7 in *yap1*^*fu48*^ mutants also rescues atrial endocardial cell numbers. A atrium, V ventricle. Scale bars, 30 μm. **j**, **k** Quantifications of endocardial cell numbers in atrium and ventricle. Mean values ± SD are shown. Two-way ANOVA was used to compare each condition with its WT control in each individual chamber (*ns* not significant; **p < 0.01;***p < 0.001; ****p < 0.0001)

The sensing and transmission of force between endothelial cells has been attributed to a mechanosensitive complex that includes Cadherin-5^20–23^. This endothelial-specific adherens junction protein links the contractile acto-myosin cytoskeleton to the cell membrane^21,24^. We wondered whether Cadherin-5 plays a similar role within endocardium and examined whether a loss of Cadherin-5 would reduce endocardial cell numbers upon the overexpression of Wnt8a or loss of Nkx2.5. Since *cadherin-5* mutants display a dysmorphic heart^24^ and lack blood flow, which would impact endocardial proliferation^4^ we used a well-established antisense oligonucleotide morpholino (MO) against Cadherin-5^25^ and selected those embryos with blood flow for further analyses. In support of an effect of Cadherin-5 on cell proliferation during chamber expansion, we found that atrial endocardial cell numbers were significantly reduced upon MO-mediated knock down of Cadherin-5 in Wnt8a overexpressing or *nkx2*.*5*^*vu179*^ mutant but not in WT hearts (Fig. 5a–f, j). Immunohistochemical analysis revealed that Cadherin-5 was localized to endocardial cell junctions in Wnt8a overexpressing or *nkx2*.*5*^*vu179*^ mutant embryos in a manner similar to WT (Supplementary Fig. 5). Within the ventricular endocardium, loss of Cadherin-5 caused diminished cell numbers (Fig. 5k), which implies that Cadherin-5 also has a physiological role in WT ventricular endocardial cell proliferation. In tune with this finding, EdU-based proliferation assays revealed that a loss of Cadherin-5 strongly diminished endocardial cell proliferation under these different conditions (Supplementary Fig. 6). To test whether this effect was due to a loss of cell junctional tensile forces upon loss of Cadherin-5, we next performed UV-laser-based force measurements (as described above) which showed that a loss of Cadherin-5 has no effect (Supplementary Fig. 7; Supplementary Movie 7). Taken together, these results suggest a role for Cadherin-5 in force sensing/transmission but not in the generation of cell junctional tensile forces within the endocardial layer during the atrial chamber expansion process.

### Yap1 is required during atrial endocardial chamber expansion

**Fig. 6:**
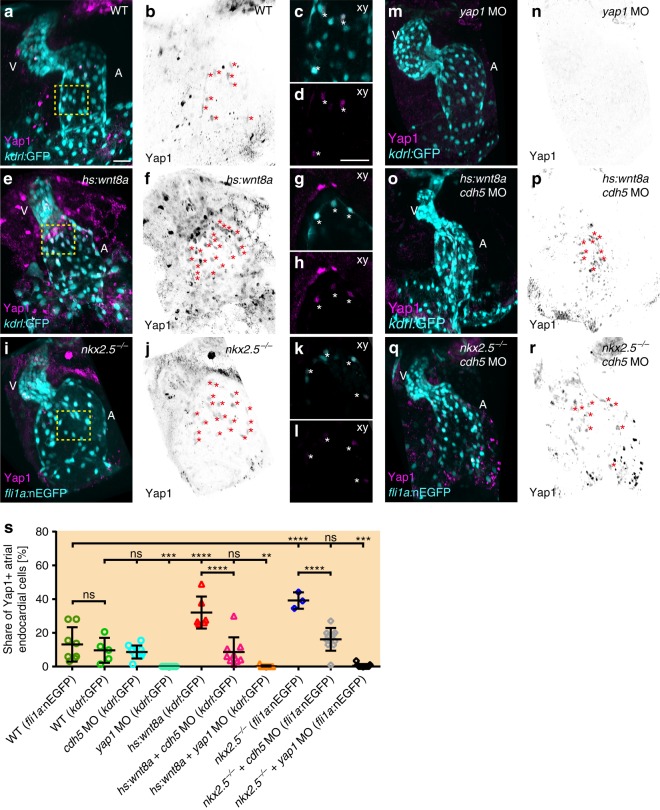
Yap1 nuclear localization within the atrial endocardium increases upon chamber expansion. **a**, **e**, **i**, **m**, **o**, **q** Reconstructions of confocal z-stacks of zebrafish hearts at 52 hpf expressing the endocardial reporters *Tg(kdrl:EGFP)*^*s843*^ or *Tg(fli1a:nEGFP)*^*y7*^ (cyan) and immunolabeling against zebrafish Yap1 (magenta). **b**, **f**, **j**, **n**, **p**, **r** Yap1 immunolabeling is inverted in black/white and endocardial cells which show co-localization with Yap1 labeling are marked with red asterisks. A atrium, V ventricle. Scale bars, 30 μm. **c**, **d**, **g**, **h**, **k**, **l** Magnifications of single confocal XY section planes (yellow box in **a**, **e**, **i**) are shown in **c**, **g**, **k** and, in **d**, **h**, **l**, only Yap1 immunolabeling is shown. Endocardial cells, labeled by *Tg(kdrl:EGFP)*^*s843*^ or *Tg(fli1a:nEGFP)*^*y7*^, that show co-localization with Yap1 are indicated with an asterisk. Scale bars, 30 μm. **s** Quantifications of the share of Yap1-positive endocardial cells relative to the total number of endocardial cells within the atrium. Upon Wnt8a overexpression (*n* = 6 hearts) or loss of Nkx2.5 (*n* = 3 hearts), the share of Yap1-positive endocardial cell numbers significantly increases within the developing atrial endocardium. Loss of Cadherin-5 (Cdh5) changes the share of Yap1-positive endocardial cells among Wnt8a overexpressing (*n* = 9 hearts) or *nkx2*.*5*^*vu179*^ mutant embryos (*n* = 10 hearts) to WT levels. Knock down of Yap1 in all conditions leads to a massive reduction of Yap1-positive endocardial cells within the atrium (*yap1* MO: *n* = 11 hearts; *yap1* MO + *hs:Wnt8a*: *n* = 9 hearts; *yap1* MO + *nkx2*.*5*^*vu179*^ mutant: *n* = 11 hearts). Mean values ± SD are shown. One-way ANOVA was used to compare each condition with its WT control (*ns* not significant, **p < 0.01;***p < 0.001; ****p < 0.0001)

Hippo signaling is involved in the control of tissue/organ size and cell proliferation in a manner that is functionally dependent on mechanical tension^26^. The Hippo pathway transcription factor Yap1 plays a role in the control of endothelial cell proliferation in response to blood flow^27,28^. We used two independent means of detecting Yap1 localization within the endocardium: First, immunolabeling with a Yap1-specific antibody revealed Yap1-positive endocardial cells within both heart chambers at 52 hpf (Fig. 6a–d; Supplementary Fig. 8a–d) which was strongly reduced in *yap1* morphants (Fig. 6m, n). Second, a transgenic Yap-specific reporter line, *Tg(fli1a:EGFP-YAP)*^*ncv35*^, which also indicated subcellular localization of Yap1 within endocardial cells (Supplementary Fig. 8e–h). To elucidate whether Yap1 functionally contributes to higher atrial endocardial cell numbers under conditions of increased tissue tension, we performed a MO-mediated knock down when Wnt8a was overexpressed or in *nkx2*.*5*^*vu179*^ mutants (Fig. 5a–c, g–i, j, k). Although knock down of Yap1 and *yap1*^*fu48*^ mutant endocardium and myocardium were morphologically indistinguishable from WT and had blood flow (Fig. 5g, j, k; Supplementary Fig. 9), a knock down of Yap1 under conditions of Wnt8a overexpression or loss of Nkx2.5, as well as the effect of the *yap1*^*fu48*^ mutation upon loss of Nkx2.5/Nkx2.7 significantly reduced endocardial cell numbers (Fig. 5j, k) and reduced atrial endocardial cell proliferation as indicated by an EdU-incorporation assay (Supplementary Fig. 6). This demonstrates an important role of Yap1 in endocardial cell proliferation control when atrial chamber dimensions strongly expand and generate tension on the tissue.

### Cadherin-5 triggers Yap1 nuclear translocation within endocardium

Increased tissue tension causes Yap1 translocation into the nucleus, where it promotes the expression of genes involved in cell proliferation^28^. E-Cadherin, for instance, was shown to induce cell cycle reentry and sequential activation of Yap1 dependent on mechanical strain^29^. We used the Yap1-specific antibody and assessed the ratio of endocardial cells with Yap1 nuclear localization relative to the entire population of atrial endocardial cells (Fig. 6). Next, we analyzed the subcellular Yap1 localization under the two conditions with increased tissue tension. We observed a significant increase in the share of atrial endocardial cells with nuclear Yap1 localization when overexpressing Wnt8a (Fig. 6e–h) or having lost Nkx2.5 (Fig. 6i–l) compared to WT (Fig. 6a–d, s). This suggests that the increase in proliferation in both genetic conditions is due to the localization of Yap1 to the nucleus.

Given the important role of Cadherin-5 in force transmission within the endocardial tissue layer, we also tested whether it was required for Yap1 nuclear translocation upon overexpression of Wnt8a or in *nkx2*.*5*^*vu179*^ mutants. Consistent with such a model, loss of Cadherin-5 reduced the share of atrial endocardial cells with a nuclear localization of Yap1 in these two conditions (Fig. 6o–s). These findings imply that Cadherin-5-mediated force sensing/transmission is required for the nuclear localization of Yap1. Another implication is that Yap1-dependent proliferation is one mode by which tensile forces within endocardial tissue are reduced during chamber expansion.

## Discussion

**Fig. 7:**
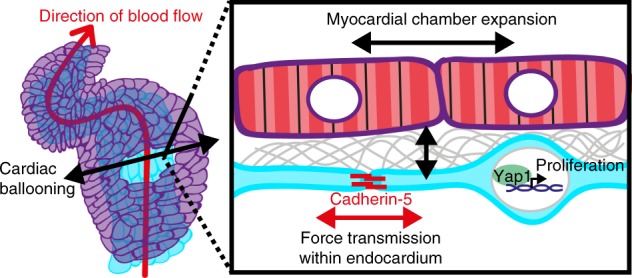
Model of intra-organ communication within the developing zebrafish heart. Myocardial chamber expansion causes increased tensile forces within the endocardial cell junctions which are transmitted by Cadherin-5. This in tune causes the nuclear translocation of the Hippo signaling transcription factor Yap1 activating cell proliferation within endocardial cells

Here, we used two different genetic conditions to model atrial myocardial chamber expansion and to study the capability of the endocardium to adapt to this enhanced myocardial growth. In summary, our work suggests that some mode of intra-organ communication between myocardium and endocardium serves to coordinate the growth of different tissues of the heart during cardiac ballooning. Our data suggests that the expansion of the myocardial chambers is transmitted to the endocardium. As a result, junctional forces rise within the endocardial tissue layer. Here, we propose a model whereby these forces are transduced via Cadherin-5 through the nuclear localization of Yap1, a transcription factor that stimulates proliferative signaling (Fig. 7). The means by which myocardial chamber expansion is communicated between the two tissue layers is currently unknown but may involve biomechanically active extracellular matrix components, which are deposited within the tight space between endocardium and myocardium^2,3^. Other means of intra-organ communication are also possible which would result in increased tissue tension within the endocardium. An alternative explanation might be that the mechanical strain due to myocardial chamber expansion is transmitted by cell junctional contacts between myocardial and endocardial cells. Just as the adaptive growth of the endocardium increases through an increase of these forces, it may end as a result of the relaxation of tensile forces on the tissue. Conversely, we also find that smaller myocardial ventricular chamber dimensions correspond with lower endocardial cell numbers. This finding suggests that this mechanism not only impacts expanding chambers but also holds true under physiological conditions of the ventricle.

The developing heart experiences many other modes of intra-organ communication based on chemical signaling^2,3^. How these diverse modes of signaling are integrated at the cellular and tissue levels remains to be addressed. The activation of proliferation within the developing endocardium in response to changes in chamber dimensions has important implications not only for our understanding of heart morphogenesis during development but also for characterizing patho-physiological conditions of the heart. It will be of great interest to revisit endocardial chamber development under conditions of congenital heart defects. The present work provides a further component in this repertoire of intra-organ communication, which causes biomechanical signaling and tensile force-triggered proliferation within the endocardium. This pattern may also hint at a general blueprint for the coordination of growth rates in the tissues of other complex organs; it shows that homeostatic responses which arise through the morphological adaptations in size of one tissue likely coordinate tissue-intrinsic growth rates within other tissues as well.

## Methods

### Zebrafish handling and lines

Handling of zebrafish was done in compliance with German, Brandenburg and Lower Saxony state law, carefully monitored by the local authority for animal protection (LAGV, Brandenburg and LANUV, Lower Saxony). The following strains of zebrafish were maintained under standard conditions as previously described^30^: *Tg(kdrl:EGFP)*^*s843*^^31^, *Tg(myl7:EGFP)*^*twu34*^ ^32^, *Tg(fli1a:nEGFP)*^*y7*^ ^33^, *Tg(flt1:YFP)*^*hu4624*^ ^15^, *Tg(hsp70l:Wnt8a-GFP)*^*w34*^ ^34^, *Tg(act2:myl12*.*1-EGFP)*^*e2212*^ ^19^, *Tg(fli1:EGFP-YAP)*^*ncv35*^ ^27^, nkx2.*5*^*vu179*^ ^12^, and *yap1*^*fu48*^^35^.

### Antisense oligonucleotide Morpholinos

Knock down studies were performed by injection of the following antisense oligonucleotide morpholinos (MO) (Gene Tools, LLC):

MO1-cadherin-5^25^: 5′-TTTACAAGACCGTCTACCTTTCCAA-3′

yap1-ATG MO^36^: 5′-AGCAACATTAACAACTCACTTTAGG-3′

nkx2.5 ATG MO^13^: 5′-TCATTTGGCTAGAGAACATTGCCAT-3′

nkx2.7 ATG MO^13^: 5′-TGGAGGTCACAGGACTCGGAAGCAT-3′

A total of 1 nl MO solution was injected into one-cell-stage embryos at the following concentration: 1 ng *yap1* MO, 0.5 ng *cdh5* MO, 4 ng *nkx2*.*5* MO, and 2 ng *nkx2*.*7* MO.

### Transgenic overexpression via heat shock

For heat shock induced overexpression of *Tg(hspl70:Wnt8a-GFP)*^*w34*^ up to 50 embryos were collected in a 2 ml Eppendorf tube and pre-warmed egg water (38 °C) was added at 24 hpf. Embryos were incubated at 38 °C for 45 min in a Thermomixer Comfort, which was turned to the side while slowly shaking at 350 rpm. After heat shock, embryos were transferred to Petri dishes filled with egg water and further incubated at 28.5 °C. At 30 hpf, GFP-positive embryos, carrying the transgenes, were selected and separated from GFP-negative fish (used as controls) and further incubated until 52 hpf.

### Immunohistochemistry

Embryos at the desired developmental stage were first anesthetized with 0.2% tricaine for one minute followed by fixation with 4% paraformaldehyde (PFA) for 1 h at room temperature. Fixed samples were washed for 30 min with PBT which was followed by blocking with 10% NGS (Normal Goat Serum) plus 2 mg per ml BSA and 0.8% Triton-X100 for 2 h at room temperature. For immunolabeling of the cell surface marker Alcam or Yap1 blocking was performed with PBDT (PBS 1× +0.1% v/v Tween 20 + 1% DMSO) supplemented with 10% NGS. The primary antibody was incubated at 4 °C overnight in PBT containing 0.8% Triton-X100. The following primary antibodies were used in this study: anti-Islet1/2 (1:10; 39.4D5, Developmental Studies Hybridoma Bank), anti-Islet1 (1:2000, Genetex, USA), anti-Alcam (1:100; zn-8, Developmental Studies Hybridoma Bank), anti-Yap1 (1:200, generated in the Lecaudey lab), anti-GFP (1:500, Aves labs, USA, #GFP-1020) and anti-atrial myosin heavy chain (1:10; S46, Developmental Studies Hybridoma Bank). After incubation samples were washed every 30 min for at least 2 h. This step was followed by secondary antibody incubation overnight at 4 °C. The following secondary antibodies were used: Alexa Fluor 647 goat anti-mouse IgG (1:250; Life Technologies), Alexa Fluor 647 goat anti-rabbit IgG (1:250; Life Technologies), and goat anti-chicken FITC (1:250, Aves labs, #F-1005). In addition, the following dyes were incubated in combination with the secondary antibody: Alexa Fluor 546 Phalloidin (1:100, Invitrogen) and DAPI (1:1000, Sigma). GFP protein within the transgenic reporter *Tg(fli1:EGFP-YAP)*^*ncv35*^ ^27^ was visualized via an anti-GFP immunolabeling. Specimens were mounted in PBT and imaged on Leica TCS SP8 confocal laser microscope.

### EdU labeling and quantification of cell proliferation

Myocardial or endocardial proliferation in embryos expressing *Tg(myl7:EGFP)*^*twu34*^, *Tg(kdrl:EGFP)*^*s843*^, or *Tg(fli1a:nEGFP)*^*y7*^ was assessed using the Click-iT EdU Imaging Kit (Life Technologies) following the previous reported protocols^37^. Anaesthetized, dechorionated embryos at 30 hpf were injected with 2 nl EdU solution (100 µM EdU, 2% DMSO, 0.1% phenol red) into the circulatory system. Incubation was performed until 52 hpf, afterwards embryos were rinsed with egg water and fixed in 4% PFA over night at 4 °C. In order to label the *Tg(myl7:EGFP)*^*twu34*^ *and Tg(kdrl:EGFP)*^*s843*^ or *Tg(fli1a:nEGFP)*^*y7*^ reporter lines a whole-mount immunostaining was performed using chicken anti-GFP (1:500, Aves labs, USA, #GFP-1020) as the primary antibody and goat anti-chicken FITC (1:250, Aves labs, #F-1005) as a secondary antibody. The Click-iT reaction for EdU staining was performed by following the manufacturer’s instructions. Images were acquired with a Leica TCS SP8 confocal laser microscope using a 20× water-immersion objective. Quantification of either proliferative myocardial or endocardial cells was performed using Imaris (Bitplane, UK). Cells were counted as proliferative when double labeled with GFP and EdU. The share of EdU-positive cells was calculated as the percentage of proliferative cells among the total number of myocardial or endocardial cells, respectively.

### Multi-color mosaic-labeling

To label the myocardium in a mosaic color fashion in vivo, myl7:TagRFP-T and myl7:TagBFP constructs were injected (kindly provided by D. Stainier)^16^ together with transposase mRNA into one cell stage transgenic *Tg(myl7:EGFP)*^*twu34*^ or double transgenic *Tg(hsp70l:Wnt8a-GFP)*^*w34*^*; Tg(myl7:EGFP)*^*twu34*^ embryos. After Tol2-mediated integration a clonal expression pattern of the constructs was created and the myocardium was labeled with a mixture of three fluorescent proteins. For live high-resolution imaging individual hearts of each condition with a colorful expression pattern were selected and mounted in 1% low-melting agarose supplemented with 0.4 mg per ml Tricaine (3-amino benzoic acidethylester; Sigma-Aldrich, A-5040) to stop the heartbeat while the images were taken. An image stack was acquired on a confocal laser scanning microscope Leica TCS SP8 with a ×20 objective every 30 min from 48–52 hpf with optical sections at 1 µm thickness. Time lapse movies and images were analyzed using Imaris Image Analysis Software (Bitplane, UK).

### Microscopy and image analysis

Images were acquired using either a Leica SP8 confocal laser microscope or a Leica SP5 confocal laser microscope with ×20 or ×25 magnification for whole mount hearts, respectively. For fixed samples, optical sections with 1 µm z-steps were generated. For live-imaging, images were taken every 30 min for up to 5 h with optical sections of 1 µm z-step thickness. Maximal intensity projections were generated using the same settings for all samples. All images were processed and analyzed using Fiji (NIH, USA) or Imaris (Bitplane, UK). Brightness and contrast were adjusted with Imaris and Fiji.

### Quantifications of Yap1-positive endocardial cell numbers

To quantify endogenous Yap1, endothelial/endocardial *Tg(kdrl:EGFP)*^*s843*^ or *Tg(fli1a:nEGFP)*^*y7*^ reporter lines were immunolabeled and DAPI was used as a nuclear marker. Cells doubly positive for nuclear Yap1 immunolabeling and an endothelial/endocardial transgene were counted as a Yap1-positive endocardial cell. The share of Yap1-positive cells was calculated as the percentage of Yap1-positive cells among the total number of endocardial cells. Images were acquired on a Leica SP8 confocal laser microscope and analyzed using Imaris (Bitplane, UK).

### Quantification of cell numbers and cell size measurements

Images were acquired on a Leica SP8 confocal laser microscope and processed with Imaris (Bitplane, UK). Maximal intensity projections were made to count the number of myocardial or endocardial cells, respectively. To count myocardial cells, the transgenic reporter *Tg(myl7:GFP)*^*twu34*^ or/and an immunohistochemistry staining against Alcam was acquired. Since the reporter line labels myocardial nuclei, anti-Alcam staining was performed to mark cell outlines. To count endocardial cells, the transgenic reporter line *Tg(kdrl:GFP)*^*s843*^ or *Tg(fli1a:nEGFP)*^*y7*^ was used. To specifically count cells within individual heart chambers, a counter staining of the myocardial atrium was performed using anti-Myosin heavy chain 6 (Myh6) immunolabeling.

Imaris was used to measure distances between two neighboring nuclei and at least 20 different length measurements were performed per chamber. Myocardial cell surface areas were quantified using Fiji (NIH, USA). Myocardial cell borders were immunolabeled with anti-Alcam. Cell surroundings were measured in Fiji using the free draw tool. Cell surface areas are displayed in μm^2^. For each heart at least 15 cells were analyzed.

### Zebrafish endocardial tissue tension measurements

Endocardial tissue tension was measured at 40 hpf using a UV-laser dissection set up as previously described^20^. First, embryos were anesthetized with 0.2% Tricaine and mounted in 1% agarose containing Tricaine to pause the heartbeat. Using a Zeiss 63 × 1.2 NA water immersion lens, 4 μm long cuts were generated with 25 pulses of 1000 Hz. Fluorescent images were acquired with a iXon DU-897-BV camera (Andor Technology) using exposure times of 200 ms and a loop time of 200 ms. To ensure comparability of the measurements, each heart was cut only once to avoid effects on the entire heart due to the release of tissue tension and only cuts without any immediate wound response were analyzed. For analysis, cells located within the lower part of the endocardial inflow tract were targeted. Within each cell, a short membrane compartment was cut which was oriented perpendicular to intra-cardiac blood flow or a long membrane compartment was cut oriented parallel to blood flow. Due to the findings that both membrane compartments show comparable initial recoil velocities, only the perpendicular membrane compartments were selected for reliable comparability. Quantification of the initial recoil velocity upon junction dissection was performed as previously described using Fiji (NIH, USA)^38,39^: A kymograph was generated to specifically display the *myl12*.*1*:eGFP signal along the junction versus time and the opening slope was determined after dissection. The tissue tension at endocardial cell junctions was calculated by linear-regression analysis of the initial relaxation velocity. For statistical analysis PRISM Graph Pad software was used.

### Statistical analyses

If not stated otherwise, all experiments in this study were performed in at least three independent biological replicates. Statistical analysis was carried out using PRISM Graph Pad software. For comparison of two groups, that were parametrically distributed, an unpaired two-tailed Student’s *t*-test was performed. In order to compare more than two groups, a one-way ANOVA test and, for two categorical independent variables, a two-way ANOVA was performed. A *p*-value of ≤0.05 was considered significant in all experiments (*p ≤ 0.05; **p ≤ 0.01; ***p ≤ 0.001; ****p ≤ 0.0001).

### Reporting summary

Further information on research design is available in the Nature Research Reporting Summary linked to this article.

## Supplementary information

Supplementary Information

Description of Additional Supplementary Files

Supplementary Movie 1

Supplementary Movie 2

Supplementary Movie 3

Supplementary Movie 4

Supplementary Movie 5

Supplementary Movie 6

Supplementary Movie 7

Reporting Summary

Source Data

## Data Availability

The authors declare that the data supporting the findings of this study are available within the paper and its supplementary information files. The rest of the data are available from the authors upon reasonable request.
