# Supplementary material for: Biomechanical signaling within the developing zebrafish heart attunes endocardial growth to myocardial chamber dimensions
Source: Nat Commun. 2019 Sep 11;10:4113. doi: 10.1038/s41467-019-12068-x (PMC6739419; doi:10.1038/s41467-019-12068-x)
Supplement: Supplementary file 1 — Supplementary Information [file 41467_2019_12068_MOESM1_ESM.docx]

**Supplementary Information**

**Biomechanical signaling within the developing zebrafish heart attunes endocardial growth to myocardial chamber dimensions**

(*Bornhorst et al.,*)

**
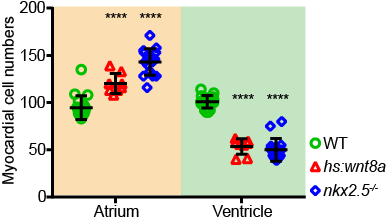
SUPPLEMENTARY FIGURES**

**Supplementary Figure 1. Overexpression of Wnt8a or loss of Nkx2.5 causes a shift in myocardial chamber proportions.** Quantifications of myocardial cell numbers reveal that atrial myocardial cell numbers are significantly increased and ventricular myocardial cell numbers significantly reduced upon Wnt8a overexpression (n=8 hearts) or in *nkx2.5^vu179^* mutants (n=16 hearts) when compared to WT (n=18 hearts). Mean values ± SD are shown. Two-way ANOVA was used to compare each condition with WT in each individual chamber (****p<0.0001).


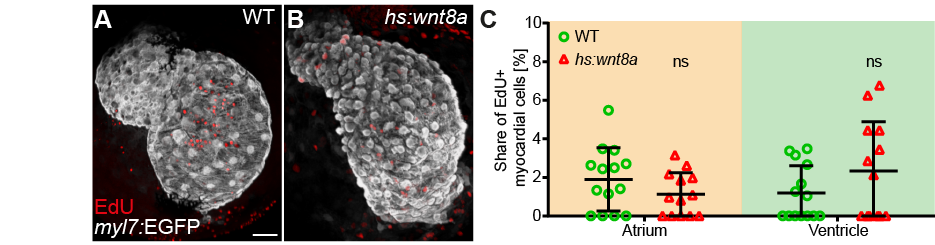


**Supplementary Figure 2. Myocardial cell proliferation does not increase upon Wnt8a overexpression. (A,B)** Reconstructions of confocal z-stacks of hearts in **(A)** WT or **(B)** Wnt8a overexpressing embryos that were injected with EdU at 30 hpf and analyzed at 52 hpf. Myocardial tissue is marked by *Tg(myl7:EGFP)^twu34^* reporter expression (white) and proliferative cells are marked by EdU incorporation (red). Scale bars, 30 µm. **(C)** Quantifications of the share of EdU+ myocardial cells relative to the total number of atrial or ventricular myocardial cells show no significant differences between WT (n=14 hearts) and Wnt8a overexpressing embryos (n=13 hearts). Mean values ± SD are shown. One-way ANOVA was used to compare each condition with WT (ns, not significant).


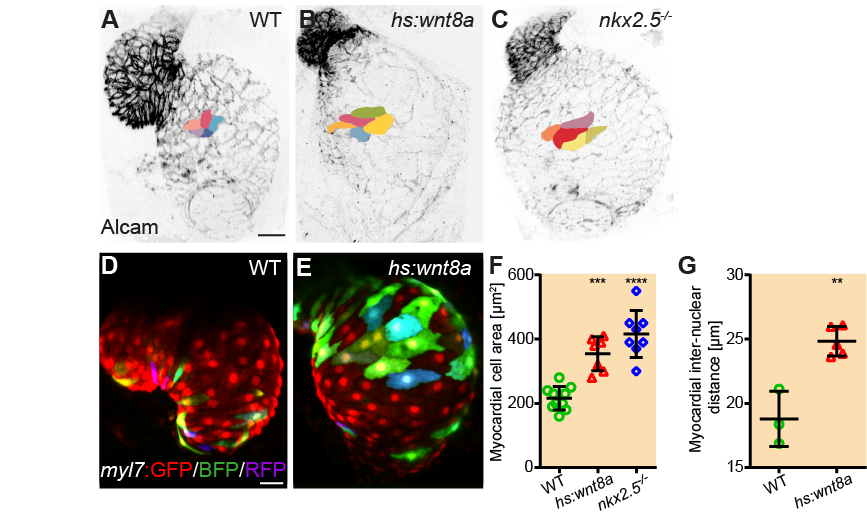


**Supplementary Figure 3. Myocardial cell surface areas increase upon Wnt8a overexpression or in *nkx2.5^vu179^*** **mutants. (A-C)** Reconstructions of confocal z-stacks of hearts at 52 hpf in **(A)** WT, **(B)** Wnt8a overexpressing embryos, or **(C)** *nkx2.5^vu179^* mutants. Myocardial cell borders are immunolabeled with anti-Alcam (shown inverted in black/white). Myocardial cell areas were measured within the myocardial atrium in all conditions and representative cells are labeled via false-coloring of cell surface areas. Scale bars, 30 µm. **(D,E)** Reconstructions of multi-color mosaically-labeled hearts at 50 hpf. Images are taken from a time lapse movies (48-52 hpf). Transgenic *Tg(myl7:EGFP)^twu34^* or double transgenic *Tg(myl7:EGFP)^twu34^*;*Tg(hsp70:wnt8a-GFP)^w34^* (false-colored red) zebrafish were injected with *myl7*:RFP-T (false-colored green) and *myl7*:BFP (blue) plasmids. Scale bars, 30 µm. **(F)** Quantifications of myocardial cell surface areas within the atrium reveal a massive increase upon Wnt8a overexpression (n=7 hearts) or in *nkx2.5^vu179^* mutants (n=8 hearts) compared to WT (n=10 hearts). Each dot represents the average of at least 15 cell surface area measurements of myocardial atrial cells. Mean values ± SD are shown. One-way ANOVA was used to compare each condition with WT (***p<0.001; ****p<0.0001). **(G)** Quantifications of myocardial inter-nuclear distances in WT (n=3 hearts) and upon Wnt8a overexpression (n=5 hearts) reveal results comparable to the measurements in (F) with massive increases in cell sizes. Each dot represents one heart with an average of at least 20 length measurements within the myocardial atrium.


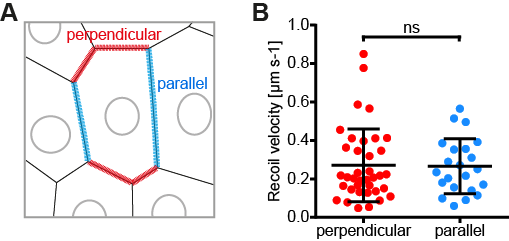


**Supplementary Figure 4. Tensile forces are comparable at perpendicular and parallel endocardial membrane compartments. (A)** Schematic model illustrating the subcortical actomyosin network within the endocardium. Laser cuts were performed either within the shorter membrane compartment which is oriented perpendicular to intra-cardiac blood flow (red) or the longer membrane compartment oriented parallel to blood flow (blue) at 40 hpf in WT. **(B)** Quantifications of the initial recoil velocities reveal no significant differences between perpendicular (n=37 hearts) versus parallel (n=22 hearts) membrane compartments after laser dissections. Mean values ± SD are shown. Students t-test was used (ns: not significant).


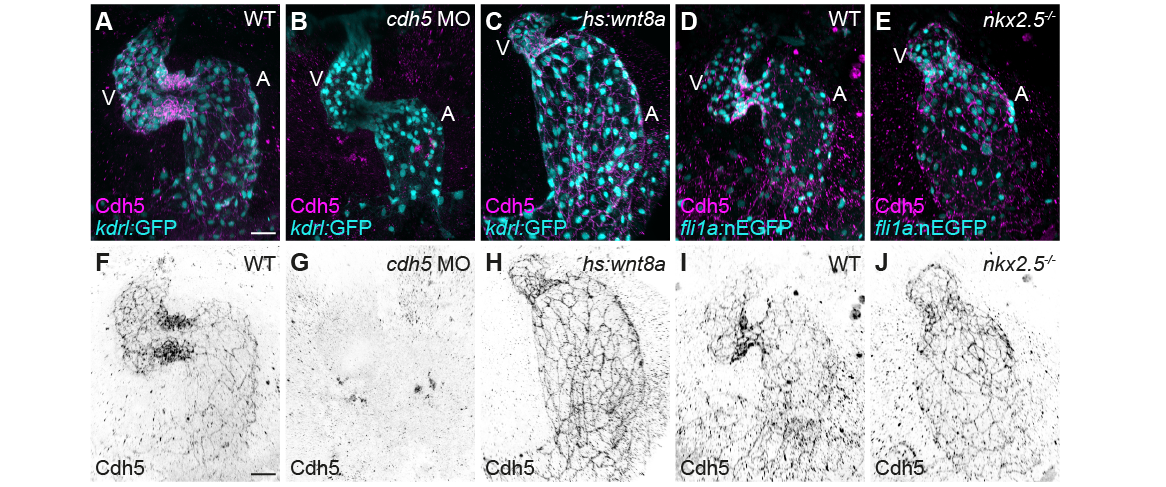


**Supplementary Figure 5. Membrane localization of Cadherin-5 upon Wnt8a overexpression or in *nkx2.5^vu179^*** **mutants. (A-E)** Reconstructions of confocal z-stacks of zebrafish hearts at 52 hpf expressing the endocardial reporters *Tg(kdrl:EGFP)^s843^* or *Tg(fli1a:nEGFP)^y7^* (cyan) and immunolabeling against Cadherin-5 (Cdh5, magenta). **(A,F;D,I)** Cdh5 is expressed and distributed at junctional membrane compartments and **(B,G)** clearly reduced in *cdh5* morphant hearts. **(F-J)** Cdh5 immunolabeling is inverted in black/white. A, atrium; V, ventricle. Scale bars, 30 μm. **(C,H,E,J)** Changes of Cdh5 expression or distribution are not visible upon Wnt8 overexpression or loss of Nkx2.5.


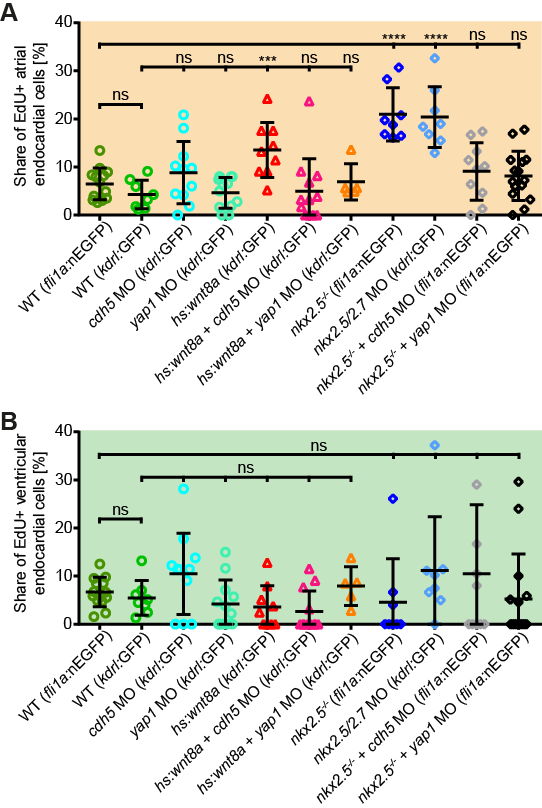


**Supplementary Figure 6. Loss of Cadherin-5 or Yap1 prevents increased endocardial cell proliferation upon loss of Nkx2.5 or overexpression of Wnt8a. (A)** Quantifications of the share of EdU-positive endocardial cells relative to the total of all atrial endocardial cells. Upon Wnt8a overexpression, in *nkx2.5^vu179^* mutants, or upon loss of Nkx2.5/Nkx2.7, the proliferation of atrial endocardial cells significantly increases. The knock down of Cadherin-5 (Cdh5) (n=11 hearts) or Yap1 (n=12 hearts) does not cause a reduction of endocardial cell proliferation within the atrium. Loss of Cdh5 upon overexpression of Wnt8a (n=12 hearts) or loss of Yap1 upon overexpression of Wnt8a (n=5 hearts) prevents increased atrial endocardial cell proliferation. Loss of Cdh5 suppresses increased atrial endocardial cell proliferation in *nkx2.5^vu179^* mutants (n=10 hearts). Loss of Yap1 via MO-mediated knock down in *nkx2.5^vu179^* mutants (n=15 hearts) also restores endocardial cell proliferation to WT levels. **(B)** Quantifications of the share of EdU-positive endocardial cells relative to all ventricular endocardial cells. Endocardial cell proliferation is not significantly different in all conditions tested. **(A,B)** Mean values ± SD are shown. One-way ANOVA was used to compare each condition with its WT control for each individual chamber (ns: not significant; ***p<0.001; ****p<0.0001).


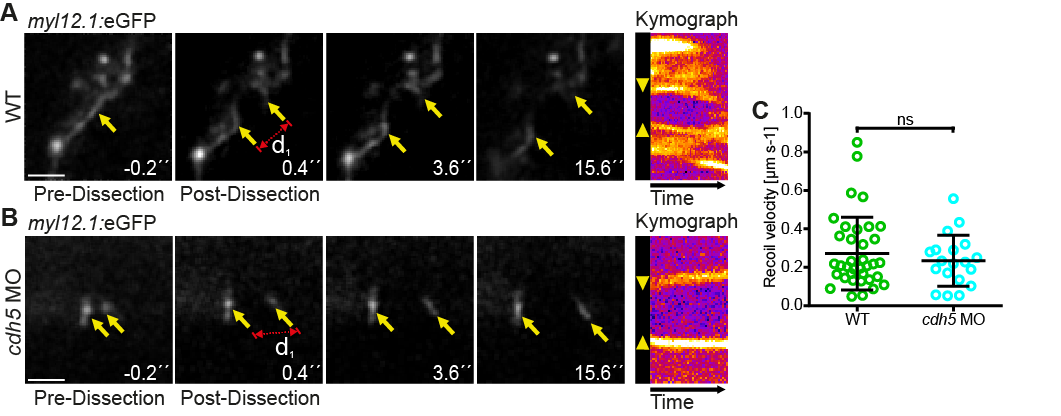


**Supplementary Figure 7. Loss of Cadherin-5 does not significantly change endocardial tissue tension. (A-B)** Time lapse sequence following a laser cut at 40 hpf within embryos carrying a *Tg(act2:myl12.1-EGFP)^e2212^* transgenic reporter that marks the actomyosin network in **(A)** control or **(B)** *cadherin-5* (*cdh5*) morphant embryos. Yellow arrows show the actomyosin recoil distance. Time lapse analyses demonstrate no significant difference in the membrane recoil upon dissection (d_1_) and no faster recoil velocities of the actomyosin network (yellow arrowheads in the kymograph) upon loss of Cdh5 compared to WT. Scale bars, 20 μm. **(C)** Comparison of initial recoil velocities (μm per sec) which are not affected in WT (n=37 hearts) compared to *cdh5* morphant embryos (n=19 hearts). Mean values ± SD are shown. Students t-test was used (ns: not significant).


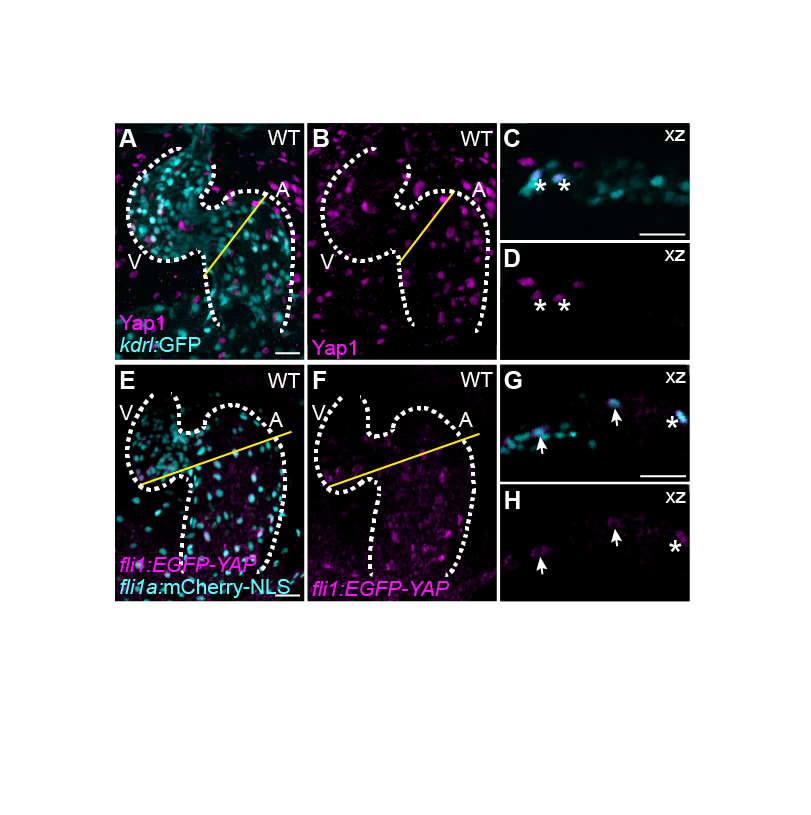


**Supplementary Figure 8. Yap1 localizes within atrial endocardial cell nuclei. (A,B)** Reconstructions of confocal z-stacks of zebrafish hearts at 52 hpf expressing the endocardial reporter *Tg(kdrl:EGFP)^s843^* (cyan) and immunolabeling against zebrafish Yap1 (magenta). **(C)** Shown is a magnification of a single confocal XZ section plane (yellow lines in A,B) or **(D)** only Yap1 immunolabeling. Endocardial cells, labeled by *Tg(kdrl:EGFP)^s843^*, that show co-localization with Yap1 are indicated with an asterisk. Scale bars, 10 μm. **(E,F)** Reconstructions of confocal z-stacks of zebrafish hearts at 52 hpf expressing the endocardial reporter *Tg(fli1a:mCherry-NLS)^ubs10^* (cyan) and the endothelial *yap1* reporter *Tg(fli1:EGFP-YAP)^ncv35^* (magenta), which shows localization of Yap1 in endothelial cells. **(G)** Shown is a magnification of a single confocal XZ section plane (yellow lines in E,F) or **(H)** only Yap1 expression. Endocardial cells, labeled by *Tg(fli1a:mCherry-NLS)^ubs10^*, show co-localization with Yap1 (asterisks). Cytoplasmic localization of Yap1 within endocardial cells is indicted with an arrow. Scale bars, 10 μm.


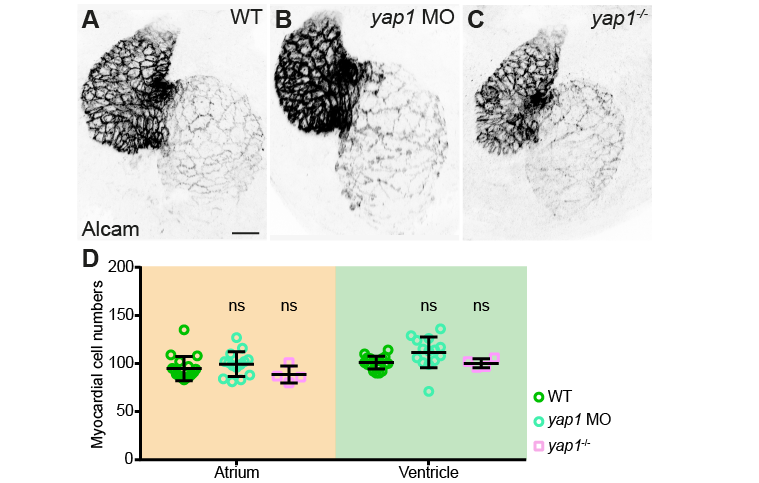


**Supplementary Figure 9. Loss of Yap1 does not alter myocardial cell numbers. (A-C)** Reconstructions of confocal z-stacks of hearts at 52 hpf in **(A)** WT, **(B)** *yap1* morphant, or **(C)** *yap1^fu48^* mutant. Myocardial cell borders are immunolabeled with anti-Alcam (shown inverted in black/white). Scale bars, 30 µm. **(D)** Quantifications of myocardial cell numbers in WT (n=18 hearts), *yap1* morphants (n=14 hearts), or *yap1^fu48^* mutants (n=4 hearts) reveals that atrial and ventricular myocardial cell numbers are not significantly different compared to WT. Mean values ± SD are shown. Two-way ANOVA was used to compare each condition with WT in each individual chamber (ns: not significant).
