# Supplementary material for: Biomechanical signaling within the developing zebrafish heart attunes endocardial growth to myocardial chamber dimensions
Source: Nat Commun. 2019 Sep 11;10:4113. doi: 10.1038/s41467-019-12068-x (PMC6739419; doi:10.1038/s41467-019-12068-x)
Supplement: Supplementary file 2 — Description of Additional Supplementary Files [file 41467_2019_12068_MOESM2_ESM.pdf]

Title: Supplementary Movie 1. Myocardial cell surface areas increase during chamber expansion in WT.
Description: Time-lapse movie based on reconstructions of a multi-color mosaically-labeled heart at 48-52 hpf. A transgenic *Tg(myl7:EGFP)*^{twu34} (false-colored red) zebrafish was injected with *myl7*:RFP-T (false-colored green) and *myl7*:BFP (blue) plasmids.

Title: Supplementary Movie 2. Myocardial cell surface areas increase during chamber expansion upon Wnt8a overexpression.
Description: Time-lapse movie based on reconstructions of multi-color mosaically-labeled heart at 48-52 hpf. Double transgenic *Tg(myl7:EGFP)*^{twu34}; *Tg(hsp70:wnt8a-GFP)*^{w34} (false-colored red) zebrafish was injected with *myl7*:RFP-T (false-colored green) and *myl7*:BFP (blue) plasmids.

Title: Supplementary Movie 3. *Tg(act2:myl12.1-EGFP)*^{e2212} marks endocardial and myocardial tissue.
Description: Confocal z-Stack sequence of a *Tg(act2:myl12.1-EGFP)*^{e2212} transgenic embryo at 40 hpf. While myocardial cells are characterized by a sarcomeric actin organization, endocardial cells display a sub-membranous actin network that is targeted during laser dissections.

Title: Supplementary Movie 4. Laser cut in a *Tg(act2:myl12.1-EGFP)*^{e2212} transgenic WT endocardium.
Description: Time series based on confocal z-stack single section planes of a *Tg(act2:myl12.1-EGFP)*^{e2212} transgenic embryo at 40 hpf. The endocardial atrial cell membrane was cut within the shorter junctional compartment and the actomyosin recoil was imaged.

Title: Supplementary Movie 5. Laser cut within *Tg(hsp70l:wnt8a-GFP)*^{w34};*Tg(act2:myl12.1-EGFP)*^{e2212} endocardium.
Description: Time series based on confocal z-stack single section planes of a *Tg(hsp70l:wnt8a-GFP)*^{w34};*Tg(act2:myl12.1-EGFP)*^{e2212} double transgenic embryo at 40 hpf. Faster recoil of the actomyosin network compared with the WT condition (Movie 4) is visible along the membrane compartment where the laser cut was performed.

Title: Supplementary Movie 6. Laser cut within a *Tg(act2:myl12.1-EGFP)*^{e2212} *nkx2.5*/*nkx2.7* double morphant endocardium.
Description: Time series based on confocal z-stack single section planes of a *Tg(act2:myl12.1-EGFP)*^{e2212} transgenic *nkx2.5*/*nkx2.7* double morphant embryo. Faster recoil of the actomyosin network compared with the WT condition (Movie 4) is visible at 40 hpf along the membrane compartment where the laser cut was performed.

Title: Supplementary Movie 7. Laser cut within a *Tg(act2:myl12.1-EGFP)*^{e2212} *cadherin-5* morphant endocardium.
Description: Time series based on confocal z-stack single section planes of a *Tg(act2:myl12.1-EGFP)*^{e2212} transgenic *cdh5* morphant embryo. The recoil of the actomyosin network along the membrane compartment where the laser cut was performed is apparently not faster compared with the WT condition (Movie 4) at 40 hpf.
